# O01 Can we reduce rates of line-associated thrombosis with routine VTE prophylaxis in OPAT patients?

**DOI:** 10.1093/jacamr/dlaf239.001

**Published:** 2026-01-05

**Authors:** Grace Barnes, Karolina Witt, Tri Wangrangsimakul

**Affiliations:** Oxford University Hospitals NHS Foundation Trust, Oxford, UK; Oxford University Hospitals NHS Foundation Trust, Oxford, UK; Oxford University Hospitals NHS Foundation Trust, Oxford, UK

## Abstract

**Background:**

Delivery of IV antibiotic therapy as part of OPAT services relies on safe vascular access. This is usually in the form of a PICC or midline, inserted prior to hospital discharge to facilitate a safe route for IV drug administration in the community. We have previously demonstrated that indwelling midlines are an independent risk factor for line-associated thrombosis (LAT) particularly in non-anticoagulated patients, suggesting we should consider VTE prophylaxis in this sub-group of our OPAT cohort.^1^ We instigated this change from December 2023 onwards.

**Objectives:**

We conducted a quality improvement project looking at the impact of VTE prophylaxis in patients discharged to the OPAT team with a vascular line in situ. We aimed to establish whether prescribing regular VTE prophylaxis for OPAT patients with a long vascular line (PICC or midline) led to a reduction in the rates of LAT in this population.

**Methods:**

We performed a retrospective review of patients under the care of the OPAT team at Oxford University Hospitals NHS Trust from January 2024 to October 2024. We generated a database (with prospective data collection completed by the OPAT nursing team during the patient’s OPAT encounter) to characterize patients with a PICC or midline, and whether VTE prophylaxis was administered. We reviewed records retrospectively to determine whether there were any episodes of LAT over this period.

**Results:**

We found that over a 10 month period, there were 181 patients under care of the OPAT team, and 150 of these patients had either a PICC or midline for antimicrobial administration (82.9%). All patients received standard care and line maintenance as per Trust policy. 111 patients with a line received some form of anticoagulation (74.0%). This included 60 patients prescribed prophylactic low molecular weight heparin specifically whilst under the OPAT team with a midline in situ (85.7% of total midlines), and patients already receiving anticoagulation for another clinical indication (e.g. 36.0% of patients with a line were already taking a regular DOAC, such as apixaban). There were only four episodes of LAT (2.6%)—these were all associated with a midline. Seventy-five percent of affected midlines were right sided, and 75% of affected patients were receiving a form of VTE prophylaxis at the time. There were no recorded adverse events directly related to VTE prophylaxis administration. We also reviewed site of line insertion as our previous audit highlighted an increased incidence of LAT in left-sided midlines. 26.0% of lines were left-sided, with only one left-sided midline LAT (25% total LATs, compared to 43.8% in previous audit).

**Conclusions:**

We demonstrate a reduction in the overall rate of LAT in our OPAT population from 7% to 2.6%, through the implementation of regular VTE prophylaxis. This highlights the role for considering VTE prophylaxis in patients who are discharged under OPAT teams with a midline in situ. Our findings may contribute to the wider body of evidence surrounding complications associated with vascular access devices and inform updates in the National OPAT Guidelines for patients at risk of LAT who may benefit from VTE prophylaxis.^2,3^
